# Foundry-enabled wafer-scale characterization and modeling of silicon photonic DWDM links

**DOI:** 10.1515/nanoph-2025-0439

**Published:** 2025-12-11

**Authors:** Robert Parsons, Alexander Oh, James Robinson, Songli Wang, Michael Cullen, Kaylx Jang, Aneek James, Yuyang Wang, Keren Bergman

**Affiliations:** Department of Electrical Engineering, 5798 Columbia University, New York, NY 10027, USA; Department of Electrical and Computer Engineering, University of Connecticut, Storrs, CT 06269, USA

**Keywords:** silicon photonics, wafer-scale photonics, DWDM links

## Abstract

AI/ML compute clusters are driving unprecedented bandwidth demands at the package boundary, motivating co-packaged integrated photonics closely co-located with the compute unit. We present a scalable silicon-photonics transceiver platform and a measurement-driven design methodology that together enable dense, energy-efficient DWDM links suitable for in-socket integration. Automated wafer-scale probing on 300 mm active photonic wafers extracts waveguide and resonator statistics using index fitting and comprehensive device characterization. The resulting wafer-scale measurements highlight design points such as wider robust waveguides, whispering gallery mode resonators, and thermally efficient undercut devices, that reduce required thermal tuning power and tighten insertion loss distributions. We propagate the measured distributions through a system model via large-scale Monte Carlo simulations to derive realistic link margins and source power targets. Together, the scalable architecture and wafer-scale measurement-informed design process offer a practical path to high-bandwidth, low energy consumption DWDM links with robust yield.

## 1 Introduction

With the extensive popularity of compute clusters in data centers for artificial intelligence and machine learning applications, the demand for higher bandwidth interconnects continues to grow. However, current interconnect solutions cannot match the performance scaling of compute resources while having significant environmental impact due to their power consumption [1], [2], [3], [4], [5]. Silicon photonics has emerged a promising platform for pushing higher data bandwidths with lower energy consumption and smaller footprint than their electrical counterparts, while being feasible at an industrial scale. By utilizing existing CMOS technology and fabrication, silicon has become the leading choice in larger-scale photonic systems [6], [7], [8], [9]. Despite the maturing fabrication process, the importance of statistical insight for device performance at the wafer-scale cannot be understated, especially for silicon photonics where non-uniformities can significantly degrade device performance [10], [11], and thus impacting link performance. Devices such as microring and microdisk resonators are the foundation of a scalable dense wavelength-division multiplexing (DWDM) photonic link, which can see significant changes in resonance location, quality factor, and modulation efficiency across a single wafer [12], [13], [14], [15], [16]. To compensate for static fabrication variations, devices are commonly integrated with micro-heaters driven by DC voltages for thermo-optic tuning [17], [18]. Hence, deviations from nominal design parameters heavily influence the energy consumption, potentially leading to inefficient operation. Additionally, resonant modulator device variations can impact the insertion loss of the device, leading to variation of associated power penalties in the link budget [19], [20]. An understanding of the statistics governing device variations allows us to model the system performance of a DWDM silicon photonic link. Here, we present a comprehensive methodology for wafer-scale measurement and data extraction of individual devices to analyze trends and correlations across 300 mm active photonic wafers. We demonstrate this in the context of a scalable architecture for co-packaged integrated photonic transceivers. Through analysis of the extracted parameters across a wafer, we estimate the expected energy efficiency of the overall link. Furthermore, we investigate insertion loss and power penalties at the wafer-scale to inform the expected variation of the link budget.

## 2 Scalable architecture

Microresonator-based DWDM transceivers driven by optical frequency combs offer compelling footprint and energy advantages for short-reach datacom [21], [22] over CW-laser arrays [23] or non-resonant modulators/filters that need extra (de-)MUX stages [24]. A common design, one bus waveguide with cascaded, slightly detuned rings, assigns each ring a wavelength while remaining transparent off-resonance [13]. The resonators are designed to have as-fabricated resonant wavelengths slightly below that of their respective comb carrier wavelengths. Integrated thermo-optic phase shifters are used to tune the modulator or filter resonant wavelength to match their carrier wavelength. Scaling breaks down once comb bandwidth exceeds the rings’ free spectral range (FSR): periodic resonances introduce resonance aliases that coincide with non-target comb lines, producing crosstalk, while packing more channels within one FSR by shrinking spacing incurs strong intermodulation; spacings below ∼100 GHz are experimentally unacceptable [25]. Increasing FSR via smaller radii helps in principle [15], [26], [27], [28] but scales poorly due to fabrication variability, tight heater/RF tolerances, and higher off-resonance loss. Band-interleaving partitions the comb into sub-bands that fit within an FSR [19], yet demands steep-roll-off, low-crosstalk, tunable elements – e.g., dichroic filters and contra-directional couplers – that are still maturing [29], [30], [31], [32], [33]. To overcome these limits, we propose an architecture that scales along two orthogonal axes: even–odd channel interleaving and multi-FSR channel allocation. Together, they support much denser combs across bandwidths far exceeding any single resonator’s FSR while maintaining low crosstalk and high efficiency – enabling massively parallel, energy-efficient optical interconnects [19].

Even–odd channel interleaving alternates comb lines into “even” and “odd” groups that are routed through separate banks of cascaded microresonators [19]. With a (de-)interleaver whose FSR is twice the comb-line spacing – and assuming ideal alignment – each de-interleaving stage halves the channel density per bus while doubling the inter-channel spacing. In practice, silicon waveguide group velocity dispersion (GVD) and fabrication variations can misalign the interleaver pass/stop bands and the comb tones. Broadband Mach-Zehnder interferometer (MZI)-based [34] (de-)interleavers – e.g., ring-assisted MZIs (RAMZIs) and MZI lattices – provide flat-top pass/stop responses, easing insertion-loss and crosstalk constraints and enabling tighter comb spacings (e.g., ≤100 GHz) while preserving the larger effective spacing seen by individual resonators [35], [36], [37], [38], [39], [40].

**Figure 1:**
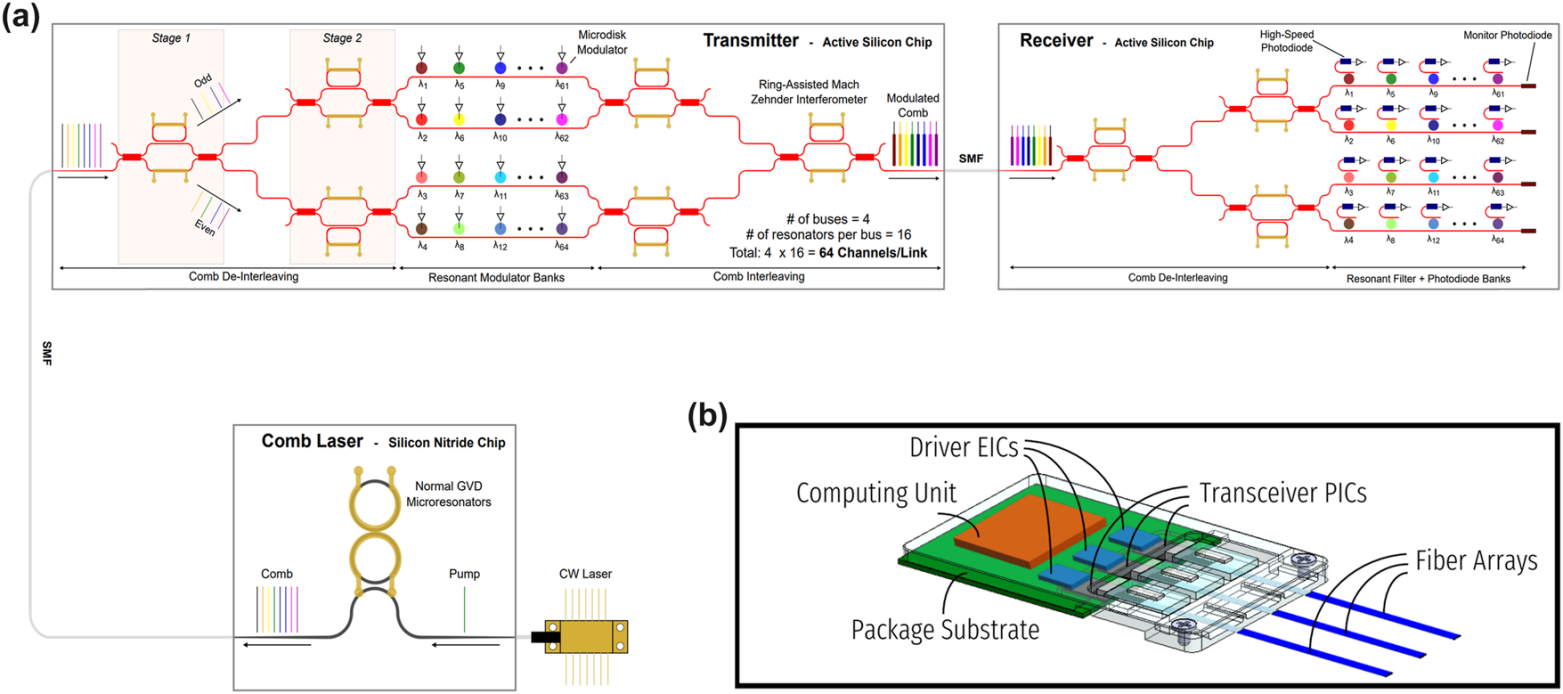
Co-packaged scalable link architecture. (a) Scalable link architecture. Normal-GVD comb laser is used as a multi-wavelength source, sent to the transmitter consisting of multiple stages of (de-)interleavers and microdisk modulators in multi-FSR regime. Encoded signals are recovered in the receiver with de-interleaver stages, microdisk filters in the multi-FSR regime, and photodetectors. (b) Conceptualization of optical transceiver utilizing scalable link architecture co-packaged with compute units in the socket.

Following even–odd de-interleaving, resonators with FSRs exceeding the retained comb bandwidth are still infeasible – small radii would be required, aggravating bend loss and fabrication tolerances [41]. We therefore adopt a multi-FSR allocation that places resonance aliases between active channels to maximize spectral isolation [19]. We define

(1a)S=ΔchΔagg,(1b)F=ΔFSRΔagg,

where Δ_ch_ is the post-de-interleaved channel spacing, Δ_agg_ is the spacing to the closest alias (“aggressor”), and FSR is the resonator free spectral range. A configuration is valid when (1) S and F are coprime integers and (2) $F\geq N_{\text{ch}}$ (channels per bus). For example, S=3 , $F=N_{\text{ch}}=7$ separates each alias by one-third of the channel spacing; smaller S enlarges Δ_agg_ but demands larger FSR, with S=1 reducing to the (often infeasible) single-FSR case. Increasing F while remaining coprime with S accommodates more channels without degrading minimum aggressor spacing. In the 4 × 16 transceiver of Figure 1a, two de-interleaving stages with S=2 , F=17 permit up to 17 channels per bus at a moderate resonator FSR of 25.69 nm; with 100 GHz comb spacing, the effective aggressor spacing is 200 GHz, a regime where inter-channel crosstalk is negligible [25], [42]. Other coprime choices (e.g., S=2 , F=19 ) offer additional channels and can be exploited using comb-envelope knowledge or thermal tuning [43]; comprehensive design trade-offs appear in refs. [19], [44].

Our approach towards a scalable implementation uses a dual-ring Si_3_N_4_ Kerr comb in normal GVD for high efficiency, uniform line power, and stable coherence [45]. Transmit-side, two cascaded RAMZI de-interleavers split the spectrum into four groups, each modulated by its own bank of cascaded microresonator modulators; a symmetric two-stage RAMZI interleaver recombines the outputs to a single fiber. The receiver mirrors this with de-interleaving, microresonator filtering, and per-channel photodiodes. With 100 GHz base comb spacing and 16 devices per bank at 400 GHz spacing, the system supports 64 DWDM channels; at 16 Gbps per channel, the aggregate capacity is 1.024 Tbps per fiber. A silicon photonic transceiver using this scalable architecture can be co-packaged directly in the socket with compute units to enable high bandwidth, low energy consumption links (Figure 1b) [46].

## 3 Results

**Figure 2:**
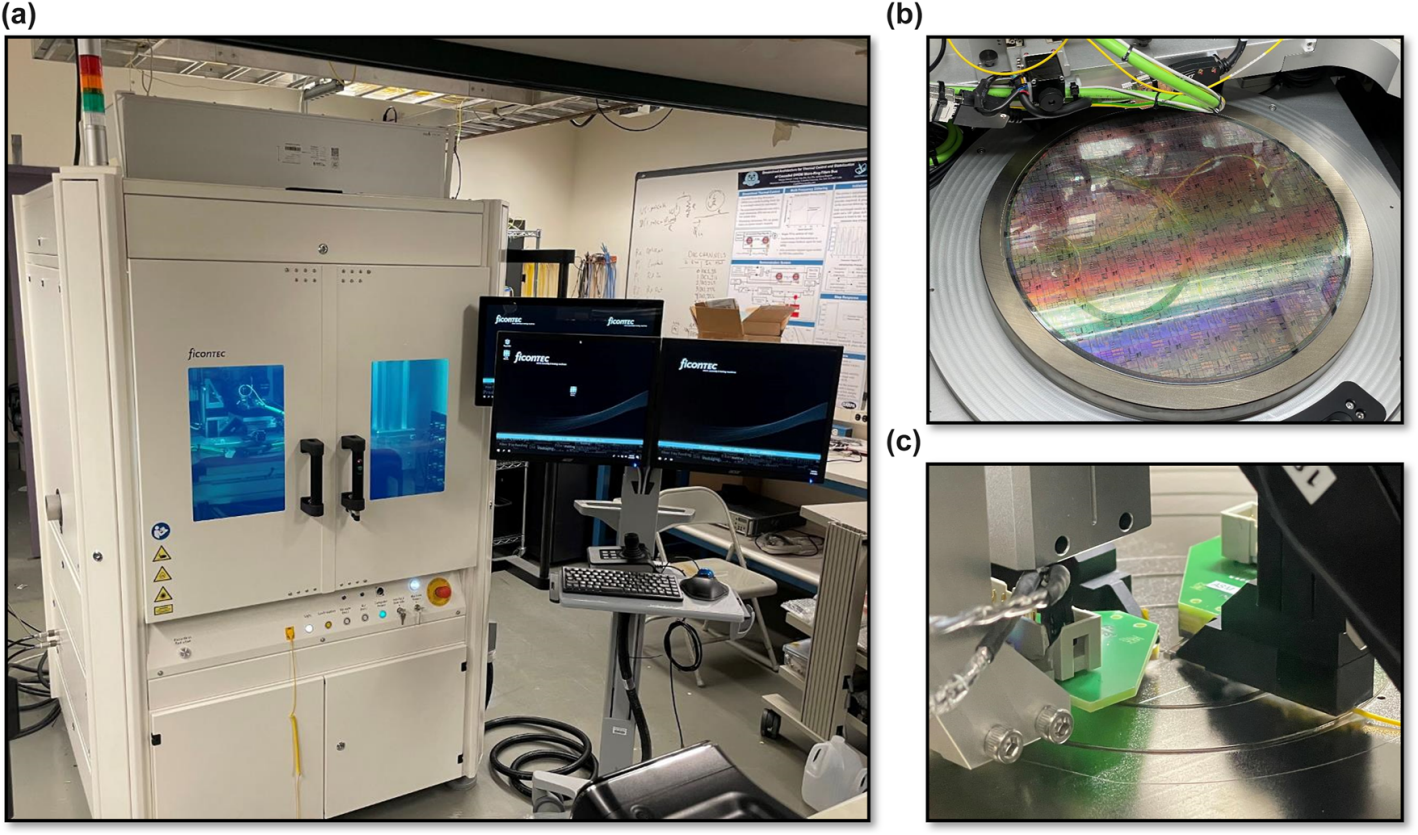
Wafer-level tester. (a) Image of ficonTEC wafer prober. (b) Image of 300 mm active photonic wafer on wafer table. (c) Image of four probes, electrical and optical, being used simultaneously on a single die.

We utilize a ficonTEC WLT-1200 wafer-level tester for automated probing of 300 mm photonic wafers [47]. Shown in Figure 2a, the wafer prober operates within an enclosure with an air filter to minimize dust and contaminants. Inside, the wafer chuck can translate, rotate, and regulate the temperature of the wafer from 20 to 80 degrees C. An image of the wafer on the wafer chuck is shown in Figure 2b. There are four probe mounts available for use – two six-axis stages on the East and West for optical probing, and two five-axis stages on the North and South for electrical probing. Both DC and RF electrical probing is possible with the wafer prober. For wafer-scale optical probing, either angled fiber arrays can be used for grating (vertical) coupling, or periscope fiber arrays can be used for edge coupling. The periscope fiber arrays utilize a 3D-printed optic with a mirror and lens that is lowered into the dicing trench which enables edge coupling at the wafer-scale. Additionally, single dies can be measured with the wafer prober, as long as the die is placed carefully over the center vacuum hole. Figure 2c displays a single die being measured with all four probes in place. Calibration of the probes is achieved with three different cameras and visual recognition techniques. Similarly, a combination of die maps generated from the layout files and visual recognition enables the wafer prober to evaluate where each device is and where to land the necessary probes. Measurement equipment is connected externally and controlled with the wafer prober’s process control master via SCPI commands over TCP/IP. Generally, a broadband tunable laser and high-precision DC power supply are interfaced with to allow measurements ranging from passive optical sweeps to thermo-optic tuning and depletion response measurements.

### 3.1 Wafer-scale optical parameter extraction

**Figure 3:**
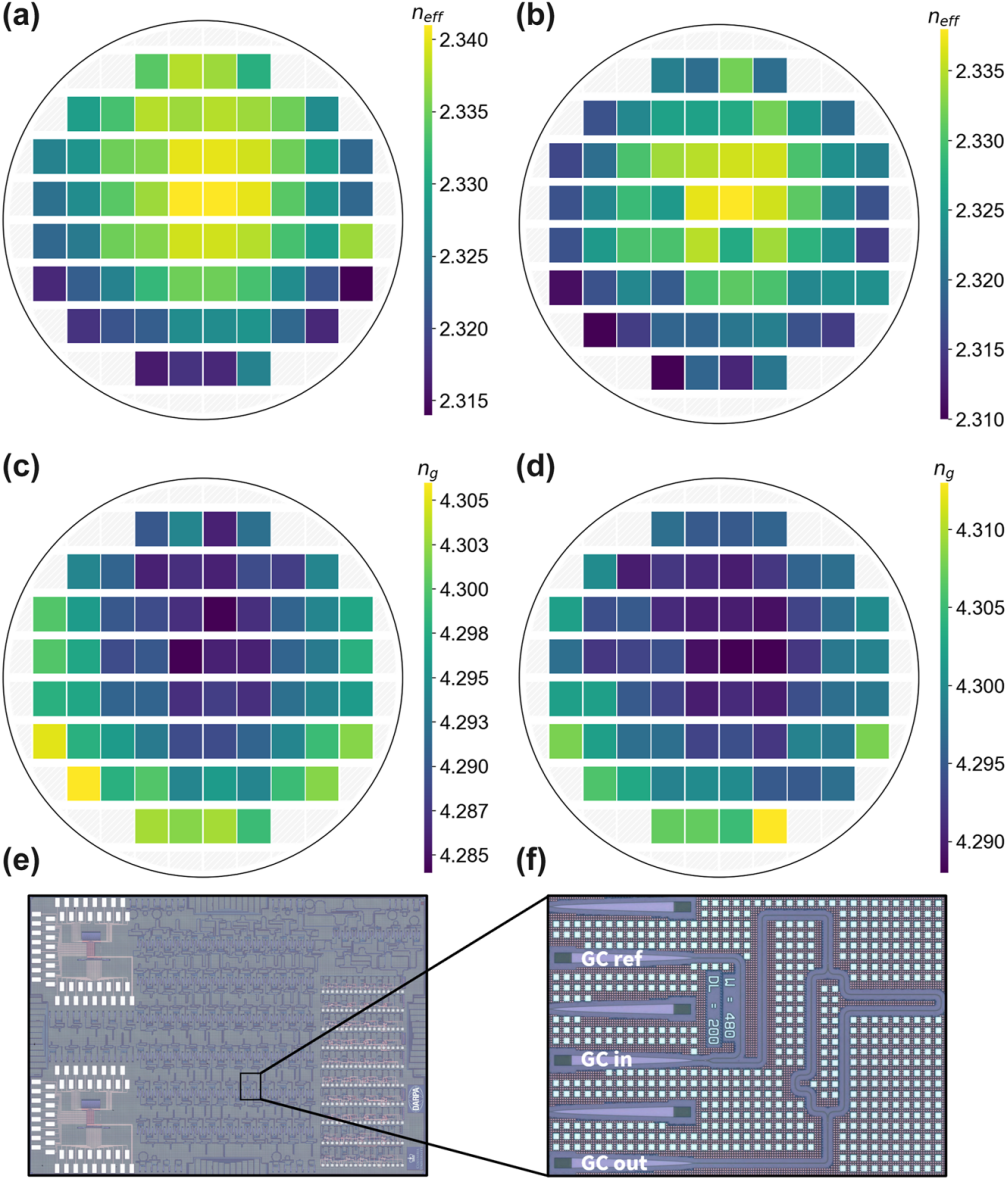
Wafer-scale characterization of waveguide effective and group index. (a) Wafermap of effective index at wavelength of 1,550 nm across wafer 1. (b) Wafermap of effective index at wavelength of 1,550 nm across wafer 2. (c) Wafermap of group index at wavelength of 1,550 nm across wafer 1. (d) Wafermap of group index at wavelength of 1,550 nm across wafer 2. (e) Micrograph of chip containing asymmetric Mach-Zehnder interferometers (AMZIs) with varying width and length imbalances. (f) Micrograph of representative grating-coupled AMZI test structure.

First, we investigate the optical properties and geometric parameters of silicon waveguides. We use a large sweep of asymmetric, or imbalanced, Mazh–Zehnder interferometers (AMZIs) with varying waveguide width and arm length imbalance (Figure 3e) across two wafers fabricated by AIM Photonics in dedicated full-reticle wafer runs. The MZIs are grating-coupled with de-embedding structures to subtract the loss envelope of the grating couplers, as shown in a representative test structure in Figure 3f. Because a large sweep of waveguide widths is used, Euler bends are employed to maintain single-mode operation within multimode waveguides; interference fringes from higher order modes could interfere with the optical parameter extraction. The transmission spectrum of each AMZI across the wafer is measured with an optical sweep using a broadband tunable laser covering the S-, C-, and L-bands. A peak-finding algorithm is used to extract the wavelength locations of each interference fringe. From here, we utilize an ordinary least squares (OLS) regression to derive fitting parameters for the group index of the AMZI following the procedure outlined in ref. [48]. Because the fringe order of the interferometer must be known to find the effective index, we must use a method to infer it. The smaller the length imbalance of the AMZI, the larger the accuracy of the inferred fringe order. A mode solver simulation sweep across waveguide width and wavelength was performed to allow us to compare the extracted group index to that of the simulated group index. By minimizing the error between these, we can find the waveguide width that corresponds to the extracted group index. The effective index at this waveguide width can then be used to infer the fringe order of the measured device, ensuring a more accurate effective index extraction.

AMZIs were measured on two wafers. We plot the wafermaps of the effective index and group index for each of the two wafers at the single mode waveguide width of 480 nm. The effective index is shown in Figure 3a and b for the first and second wafer, respectively, and the group index is shown in Figure 3c and d for the first and second wafer, respectively. It is clear that there is a radial relationship shown in each of the four wafermaps. To demonstrate this statistically, we mapped the locations of the reticles to polar coordinates in reference to the center of the wafer and performed an OLS regression. The correlations for radial relationships for effective index were 0.507 and 0.623 for each wafer, respectively, and for group index, 0.527 and 0.502, respectively. As suggested by the slanted bullseye appearance of the wafermaps, there also exists a planar relationship. Again, we performed an OLS regression, this time using Cartesian coordinates and projecting across the wafer at varying angles. This way, we understand not only the strength of the planar relationship, but also the angle at which the plane is situated. The correlations and angles for planar relationships for effective index were 0.351 (264.15 deg) and 0.174 (245.18 deg) for each wafer, respectively, and for group index, 0.355 (259.72 deg) and 0.291 (258.72 deg), respectively. These trends could be explained by temperature variations in the process.

**Figure 4:**
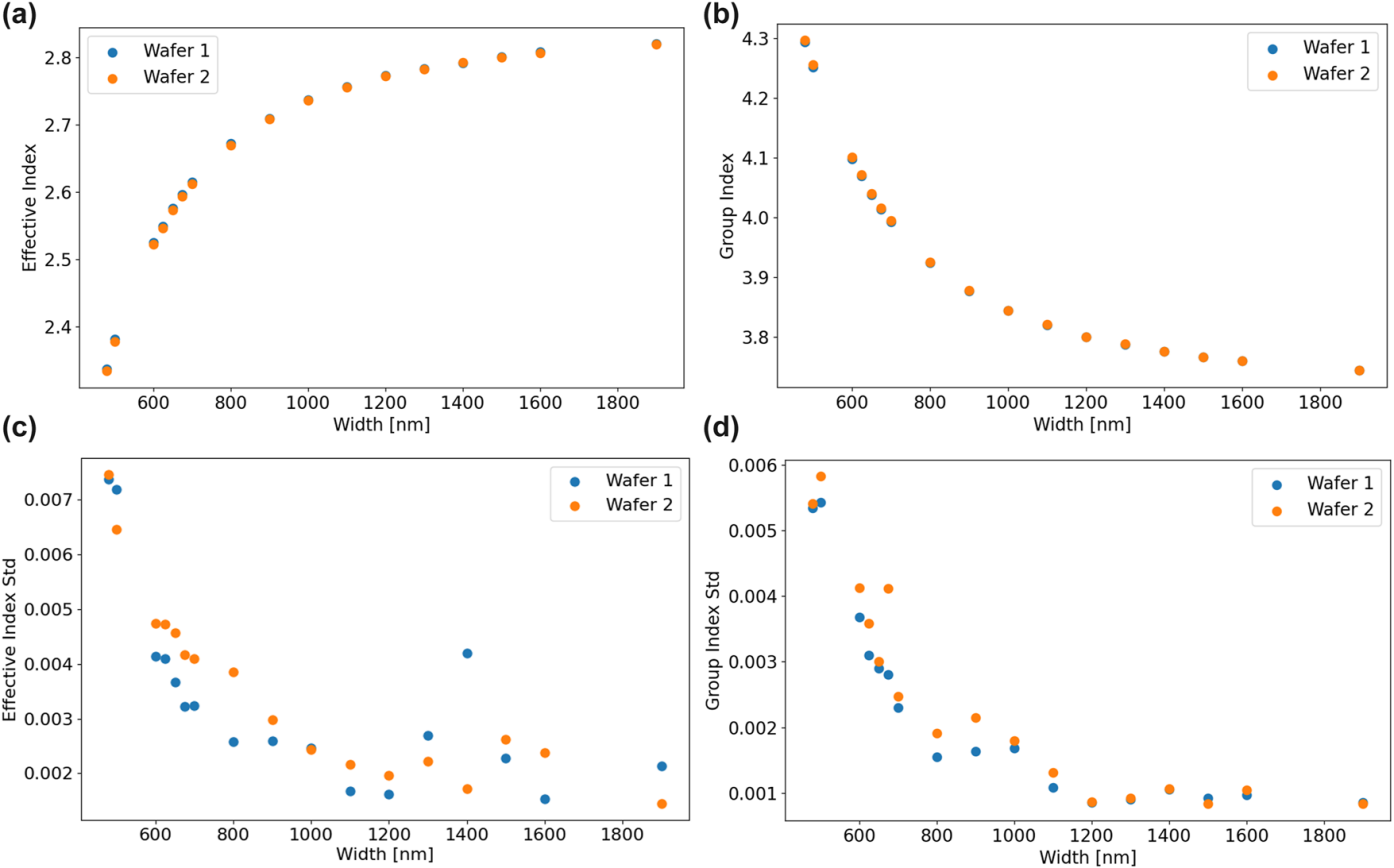
Effective and group index statistics across waveguide width. (a) Effective index averaged across each wafer at different waveguide widths at wavelength of 1,550 nm. (b) Group index averaged across each wafer at different waveguide widths at wavelength of 1,550 nm. (c) Standard deviation of effective index averaged across each wafer at different waveguide widths. (d) Standard deviation of group index averaged across each wafer at different waveguide widths.

The resulting extracted wafer-scale optical parameters are displayed in Figure 4. The effective and group indices averaged over the 64 reticles across each wafer are shown in Figure 4a and b, respectively. The effective and group indices are calculated across waveguide widths supporting only a single mode up to those supporting many higher order modes. As expected, both parameters head toward an asymptote at highly multimode waveguide widths, as their behavior starts to exhibit that of a slab mode. The high consistency between wafers is noteworthy for each parameter. Additionally, the standard deviation for both the effective index and group index is displayed in Figure 4c and d, respectively. There is a clear trend that larger waveguide widths experience drastically reduced statistical variation in both effective index and group index. This is explained by the mode being less perturbed proportionally by etching biases due to the much larger width. Further, the mode of larger waveguides is more confined and is less affected by changes in sidewall. There is relatively low variation between the two measured wafers, despite the two wafers being from different lots. We expect wafer-to-wafer variations to be lower within the same lot compared to variability between wafers from different lots.

**Figure 5:**
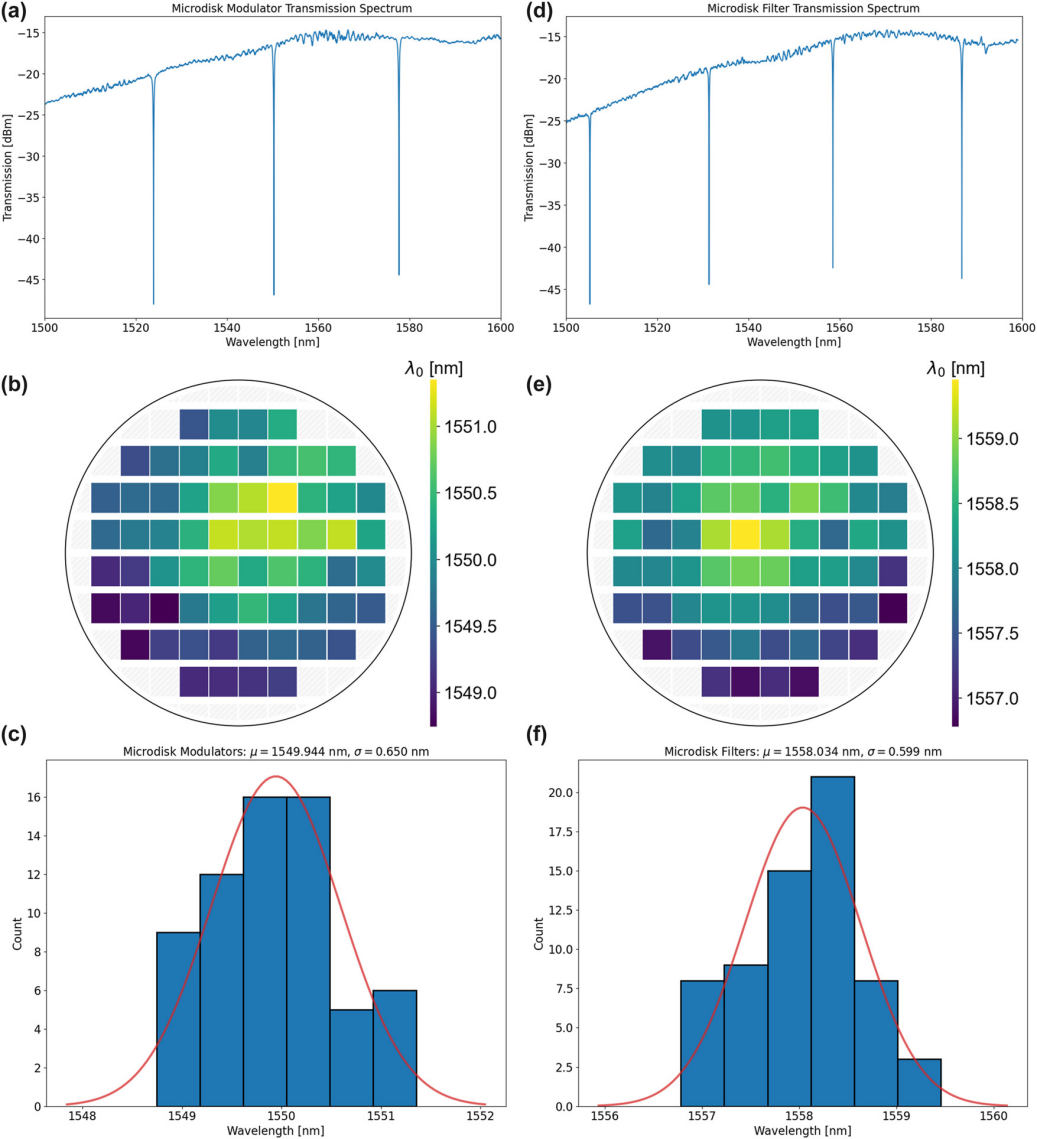
Wafer-scale microresonator characterization. (a) Transmission spectrum of a microdisk modulator. (b) Wafermap of microdisk modulator resonance location across the wafer. (c) Histogram and statistical data of microdisk modulator. (d) Transmission spectrum of a microdisk filter. (e) Wafermap of microdisk filter resonance location across the wafer. (f) Histogram and statistical data of microdisk filter.

We measured microdisk modulators and filters on our latest 300 mm wafer taped out with AIM Photonics. The transmission spectrum of a representative microdisk modulator is shown in Figure 5a, the wafermap displaying the resonance locations across the wafer is shown in Figure 5b, and a histogram of the distribution is displayed in Figure 5c. Similarly, the transmission spectrum, wafermap, and histogram for the microdisk filter are shown in Figure 5d–f, respectively. The variation of the modulator is slightly higher than that of the filter (standard deviation of 0.65 nm vs. 0.6 nm) due to the modulator junction width being slightly smaller to reduce series resistance and factoring in variations of the doped junction in the modulator. However, both demonstrate high robustness to variations compared especially to microring resonators. Microrings of a similar radius demonstrated standard deviations on the order of multiple nanometers, further solidifying these as robust devices.

### 3.2 Implications for link energy consumption

With a suite of devices now measured and analyzed at the wafer-scale, we can use these results to calculate power consumption to inform component and link design (Table 1). The variation of effective index, extracted from the AMZI test structures, can be converted mathematically to waveguide phase error. This phase error due to fabrication variations must be compensated for by thermal tuning of the device. In our scalable link architecture, the devices that are sensitive to these waveguide phase errors are the interleavers. Therefore, we can calculate the required thermal tuning per thermo-optic phase shifter within the interleaver. Accounting for 3*σ* variation allows us to cover about 99.7 % of all devices, statistically speaking. From the effective index standard deviation of 480 nm wide waveguides, we calculate a 3*σ* phase error of 7.887 radians. Assuming a tuning efficiency of 25 mW *P*_*π*_, on average 31.38 mW would be required. However, as we noted previously, moving to a larger waveguide width reduces the variation of optical parameters significantly. Therefore, using wider waveguides in the interleavers enables higher robustness to fabrication variations and an associated reduced energy consumption. Using the same tuning efficiency, 1,200 nm wide waveguides would exhibit a 3*σ* phase error of 2.154 radians, leading to an average tuning power of 8.57 mW. This is a significant reduction. Moreover, the 3*σ* phase error being less than *π* enables easier alignment to channels. Moving on to the microdisks, using the numbers reported in Figure 5 and the device FSR [49], we have 3*σ* phase errors of 0.451 and 0.416 radians for the microdisk modulators and filters, respectively. This leads to an associated average tuning power of 5.91 and 2.46 mW, respectively. The filter exhibits a larger tuning efficiency due to no RF contact vias and closer placement to the optical mode. Using a thermal undercut of the substrate significantly improved the thermal tuning efficiency of devices [50]. If we apply those improvement factors here to these already robust components, then the average tuning power required would now be 1.34 mW, 0.37 mW, 1.18 mW, and 0.49 mW for the 480 nm waveguide phase shifter, 1,200 nm waveguide phase shifter, microdisk modulator, and microdisk filter, respectively. The resulting power consumption that each component contributes to the link is calculated and displayed in Table 1. Clearly, utilizing robust component designs and strategies to improve tuning efficiency, such as thermal undercut, are imperative to reducing the power consumption of links due to thermal tuning requirements.

**Table 1:** Energy consumption of link components due to thermal tuning requirements.

Component	3*σ* phase error [rad]	Power [mW] (nominal)	Power [mW] (undercut)
Interleaver (480 nm)	7.887	31.38	1.34
Interleaver (1,200 nm)	2.154	8.57	0.37
Microdisk modulator (4 μm)	0.451	5.91	1.18
Microdisk filter (4 μm)	0.416	2.46	0.49

### 3.3 Wafer-scale link parameter extraction

**Figure 6:**
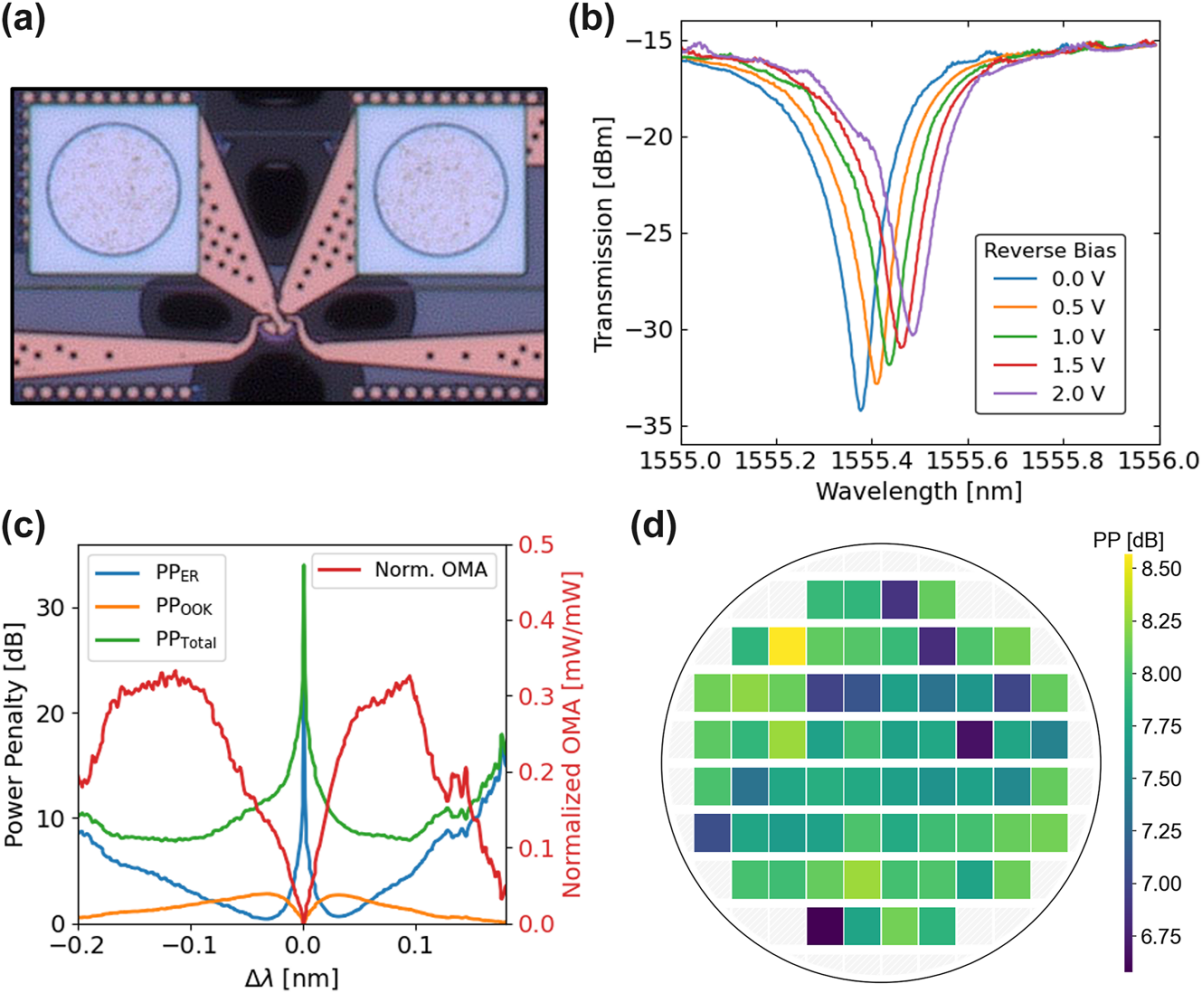
Microdisk modulator power penalties. (a) Micrograph of a microdisk modulator. (b) Depletion response of microdisk modulator demonstrating changing spectrum with varied applied reverse bias to the junction. (c) Power penalties, insertion loss, and optical modulation amplitude of modulator as function of detuning wavelength. (d) Wafermap of total power penalty of modulators in each reticle.

First, we look at one of the primary contributors to the link loss budget – the power penalties associated with modulation. Important to understanding the power penalties associated with the resonant modulator, shown in Figure 6a, is the depletion response of the device (Figure 6b). The insertion loss (IL) and extinction ratio (ER) vary depending on the driving voltage of the modulator and carrier detuning from the resonant wavelength. As shown in Figure 6c, the IL, ER, and associated power penalties can be calculated based on the detuning of the carrier from the resonant wavelength. The total power penalty is given by the following equation:

(2) $${PP}_{\text{total}}={IL}_{\text{mod}}+{PP}_{\text{ER}}+{PP}_{\text{OOK}}$$

where

(3a)PPER=10log10r+1r−1,(3b)PPOOK=10log102rr+1,

and *r* is the linear extinction ratio. As the extinction ratio increases and approaches infinity, the power penalty associated with the ER approaches zero, while the power penalty associated with on–off keying approaches 3 dB (equal distribution of ‘1’s and ‘0’s), for 3 dB total. There are diminishing returns of power penalty after achieving an ER of at least 10 dB. A large depletion response is necessary for a small insertion loss while maintaining a low driving voltage. In addition, the optical modulation amplitude (OMA) is shown to be maximal at the minimum of the total power penalty; the OMA normalized to the input power is defined as

(4) $${OMA}_{\mathrm{norm}}=\left(P_{1}-P_{0}\right)/P_{\text{in}},$$

where *P*_1_ is the ‘on’ state optical power, *P*_0_ is the ‘off’ state optical power, and *P*_in_ is the input optical power. With the ability to calculate these power penalties from the depletion response, we measured the depletion response of disk modulators across a wafer. The calculated total power penalty, including OOK and ER penalties and IL, is shown in the wafermap in Figure 6d. With a driving voltage of only 1 V, the mean total power penalty is 7.78 dB, with a standard deviation of 0.41 dB. Since the resonant modulators are critically coupled, fabrication variations can cause a moderate change in the extinction ratio, resulting in the larger standard deviation. However, moving toward resonant modulators that are slightly over-coupled will help to reduce this standard deviation, as the extinction ratio of over-coupled modulators are less sensitive to fabrication variations.

**Figure 7:**
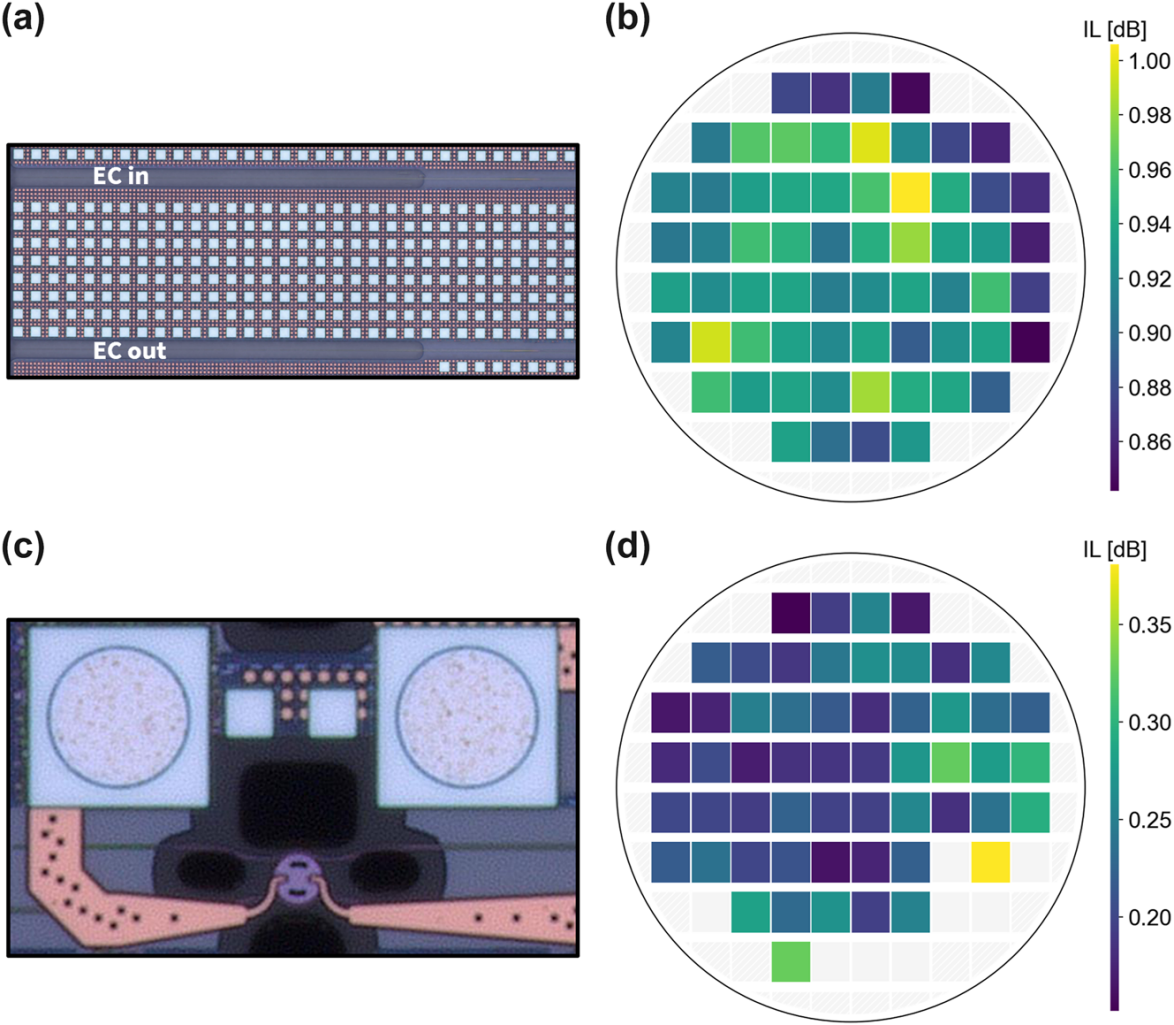
Wafer-scale insertion loss of edge couplers and microdisk filters. (a) Micrograph of an edge coupler with substrate removed underneath. (b) Wafermap of per-facet coupling losses between edge couplers and cleaved SMF28. (c) Micrograph of a microdisk filter. (d) Wafermap of insertion loss seen at the drop port of add-drop microdisk filters.

Coupling between fiber and chip has traditionally also been a challenge in terms of contending with excess losses [51], [52], [53]. Although grating couplers are available, we use edge couplers for both higher coupling efficiency and larger optical bandwidth, important for broadband DWDM links. A representative edge coupler is shown in Figure 7a. The substrate below the silicon nitride edge coupler is partially removed and backfilled with silicon dioxide. This dramatically improves the mode matching between the cleaved SMF28 mode and that of the silicon nitride waveguide, thereby dramatically improving the possible coupling efficiency. Low-loss and broadband escalators enable coupling between silicon nitride and silicon device layers. Using a periscope fiber array, we obtained wafer-scale per-facet coupling losses by measuring a loopback structure in each of the 64 reticles. The loss per coupler is shown in Figure 7b, with a mean coupling efficiency of 0.924 dB and standard deviation of only 0.036 dB. These sub-dB edge couplers demonstrate high robustness to variations in both fabrication and positioning of the fiber array (similar process to that used when attaching the fiber array), critical to realizing ultra-broadband links.

On the receiver side, wavelength-selective resonant filters must be used to filter out and drop each encoded carrier. Assuming a large enough filter bandwidth (or alternatively, lower quality factor) is used to avoid attenuating parts of the signal, the main parameter of interest is the insertion loss seen at the drop port of the device. If critical coupling is not achieved, the insertion loss can become quite high, straining the link loss budget. We utilize microdisk filters for their robustness to fabrication variations (Figure 7c). Another advantage is that the cavity loss is small, owing to the supported whispering gallery mode. This allows a simple symmetric coupling scheme to produce low insertion loss add-drop filters; a wafermap of the insertion loss seen at the drop port of microdisk filters is shown in Figure 7d. The mean and standard deviation insertion loss is 0.225 dB and 0.047 dB, respectively. Introducing a slight asymmetry to the coupling can reduce this loss even further.

### 3.4 Implications for link optical power budget

**Figure 8:**
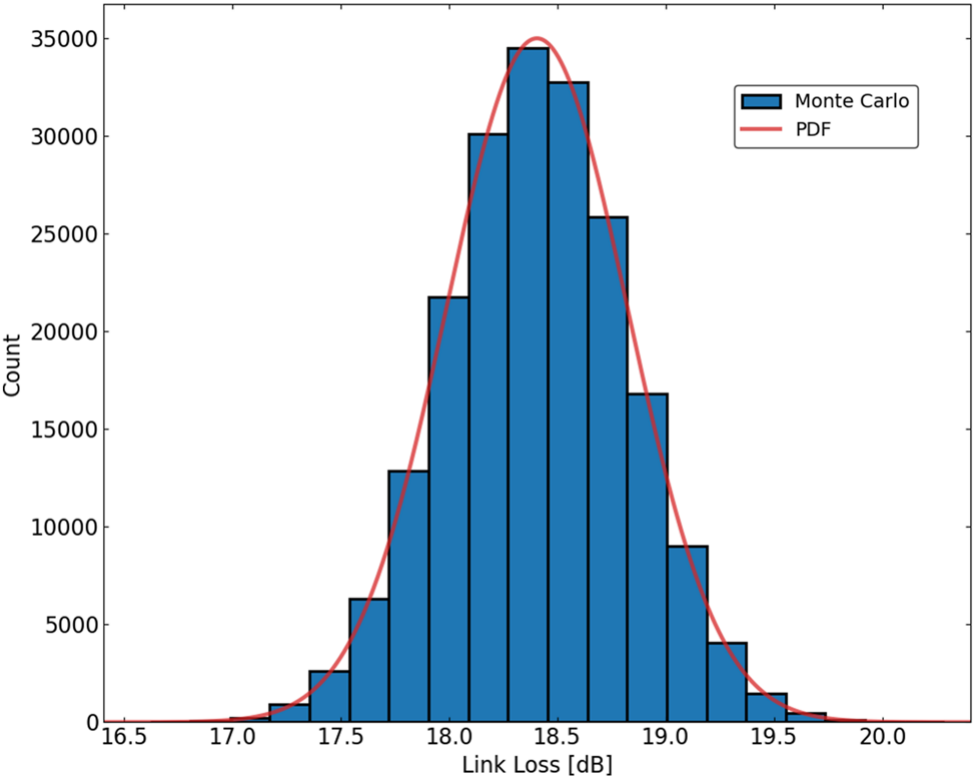
Monte Carlo simulations (*n* = 200,000) showing total link loss incorporating statistical variations of components from wafer-scale measurements. Mean and standard deviation link losses were 18.407 dB and 0.418 dB, respectively.

The various insertion losses and power penalties of each component within the link measured from test structures are tallied in Table 2. Statistical information is included where mass-measurement was possible, however all values are based on measured data. To better understand the system-level implications of component-level variability in integrated photonic links, we perform a Monte Carlo simulation using the available statistical data obtained from wafer-scale measurements. While one-off measurements provide estimates of link loss, they do not capture the full distributional behavior of the link budget. A Monte Carlo approach enables us to propagate measured variations, including insertion losses, coupling efficiencies, and modulation power penalties, through the full link architecture. This allows for a more accurate estimation of performance metrics such as link yield for a given power budget and worst-case design margins (e.g., 3*σ* link loss). Using the available wafer-scale measurements, 200,000 Monte Carlo simulations were performed on the link budget (Figure 8). The resulting mean link loss was 18.407 dB, and the corresponding standard deviation was 0.418 dB. Given a receiver sensitivity for error-free operation at 16 Gbps per channel [49], incorporating the 3*σ* link loss into our tabulated link budget as the margin allows us to describe the expected behavior of 99.7 % of links (Table 2). By statistically sampling from the known distributions of each component, we gain insights into opportunities for targeted yield improvement through component-level optimization.

**Table 2:** Link loss budget with tabulated per-component losses, including statistical information where available.

Loss component	Value	1*σ*	Unit	Multiplier	Source
**Transmitter loss total**	**15.06**	**–**	**dB**		
Comb to fiber	2.20	–	dB		Measured
Edge coupler in	0.924	0.036	dB		Measured
De-interleaver stages	0.35	–	dB	×2	Measured
Modulator off-resonance	0.10	–	dB	×15	Measured
Modulator power penalties	7.78	0.41	dB		Measured depletion response
Crosstalk penalty	–	–	dB		>100 GHz channel spacing
Interleaver stages	0.35	–	dB	×2	Measured
Propagation loss	0.33	–	dB		PIC layout
Edge coupler out	0.924	0.036	dB		Measured
**Receiver loss total**	**3.35**	**–**	**dB**		
Edge coupler in	0.924	0.036	dB		Measured
De-interleaver stages	0.35	–	dB	×2	Measured
Filter off-resonance	0.10	–	dB	×15	Measured
Filter on-resonance	0.225	0.047	dB		Measured
Crosstalk penalty	–	–	dB		>100 GHz channel spacing
**Link loss total**	**18.41**	**–**	**dB**		
+ Margin	1.253	–	dB		3*σ* link loss variation
+ Receiver sensitivity	−22.37	–	dBm		For 16 Gbps/channel
**= Min. power per line**	**−2.71**	**–**	**dBm**		

## 4 Conclusions

We have presented a wafer-scale, measurement-driven framework that links device-level statistics to system-level performance for scalable, co-packaged silicon photonic interconnects. Building on a scalable, comb-driven DWDM architecture that combines even–odd channel interleaving with multi-FSR channel allocation, we quantified the impact of fabrication variability across 300 mm wafers and propagated these statistics through a realistic system model. The resulting methodology connects waveguide and resonator non-uniformities to thermal tuning power requirements, power penalties, and end-to-end link margins. Our wafer-scale extraction of effective and group indices revealed strong radial and planar trends, and demonstrated that wider waveguides substantially suppress statistical variation, thereby reducing required thermo-optic tuning power in phase shifters. Microdisk modulators and filters exhibited tight resonance distributions and low insertion losses at scale, while per-facet edge coupling losses remained sub-dB with minimal spread. Incorporating the measured depletion responses, we mapped modulation power penalties, and then executed a 200,000-trial Monte Carlo analysis of the full link. The resulting mean link loss (18.41 dB) and variation (0.418 dB) translate into clear source power targets when combined with receiver sensitivity and a 3*σ* margin. Importantly, design choices identified here–wider interleaver waveguides, whispering gallery mode resonators for both modulators and filters, and thermal undercut for improved heater efficiency–drive tuning power reductions to improve energy per bit. These results validate the proposed architectural path for dense, comb-driven DWDM. The combination of measured device distributions and system-level Monte Carlo yields a practical path forward for scaling channel count and aggregate bandwidth while maintaining low energy consumption for in-socket, co-packaged integrated photonics.
